# Supplementary material for: The interactome of intact mitochondria by cross-linking mass spectrometry provides evidence for coexisting respiratory supercomplexes
Source: Mol Cell Proteomics. 2017 Dec 8;17(2):216–32. doi: 10.1074/mcp.RA117.000470 (PMC5795388; doi:10.1074/mcp.RA117.000470)
Supplement: Supplemental Data [file supp_RA117.000470_133922_0_supp_23978_fzffwf.zip › spectra_annotation/mito_spectra_annotation/247-1-5-1-12-1.pdf]

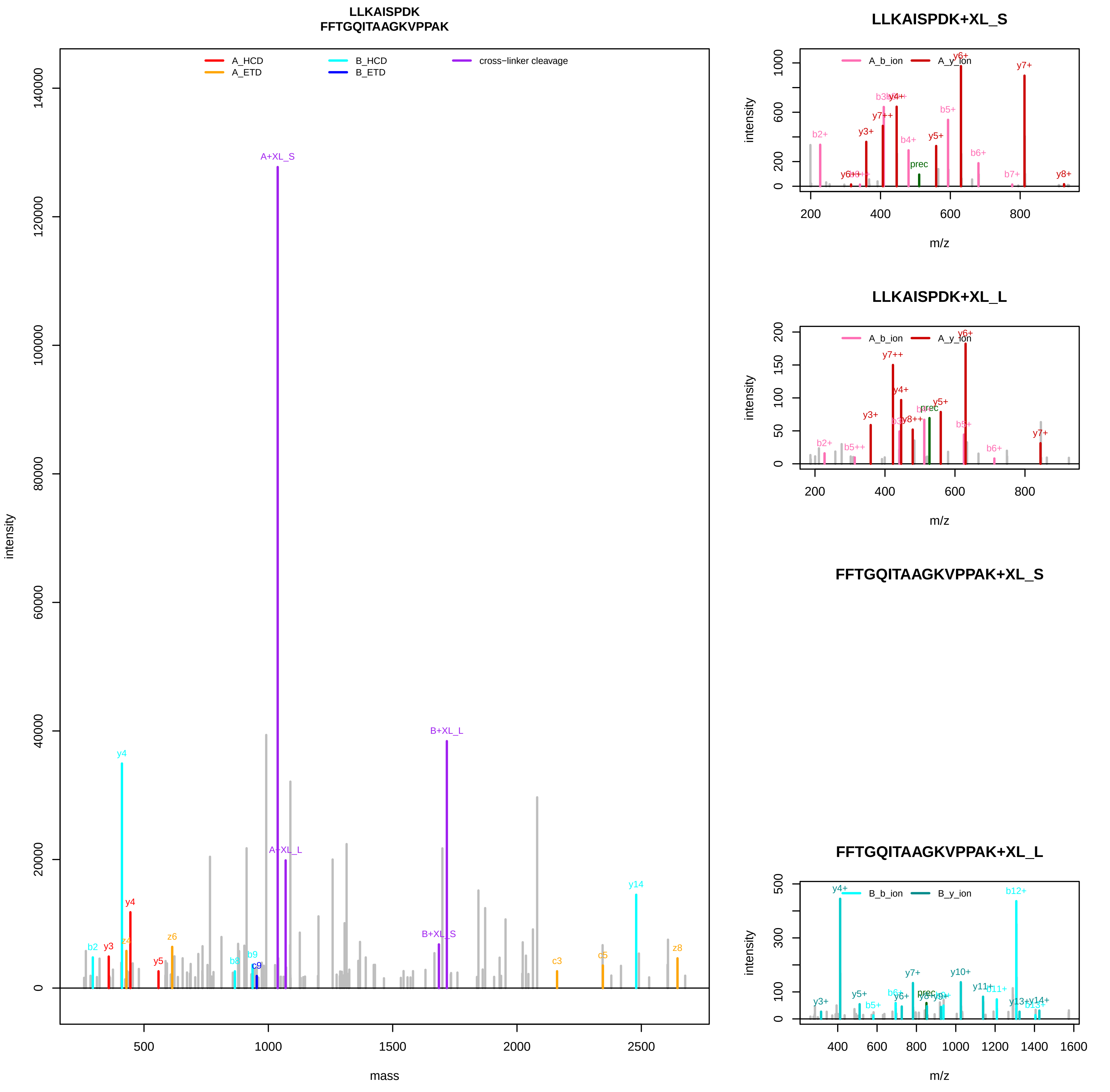
LLKAISPDK
FFTGQITAAGKVPPAK
A_HCD
A_ETD
B_HCD
B_ETD
cross−linker cleavage
A+XL_S
A+XL_L
B+XL_L
B+XL_S
b2
y3
y4
z4
y5
z6
b8
b9
c9
c3
c5
y14
z8
intensity
mass
0
20000
40000
60000
80000
100000
120000
140000
500
1000
1500
2000
2500
LLKAISPDK+XL_S
A_b_ion
A_y_ion
b2+
y3+
y7++
b4+
b5+
y5+
y6+
y7+
b6+
b7+
y8+
prec
intensity
m/z
LLKAISPDK+XL_L
A_b_ion
A_y_ion
b2+
b5++
y3+
y7++
y4+
y8++
prec
y5+
b5+
y6+
b6+
y7+
intensity
m/z
FFTGQITAAGKVPPAK+XL_S
FFTGQITAAGKVPPAK+XL_L
B_b_ion
B_y_ion
y3+
y4+
y5+
b5+
y6+
y7+
prec
y10+
y11+
b11+
b12+
y13+
y14+
b13+
intensity
m/z
